# A novel Hoxd13 mutation causes synpolydactyly and promotes osteoclast differentiation by regulating pSmad5/p65/c-Fos/Rank axis

**DOI:** 10.1038/s41419-023-05681-8

**Published:** 2023-02-20

**Authors:** Lishan Zhang, Ziqi Fang, Guangdong Cheng, Mengting He, Yanliang Lin

**Affiliations:** 1 Department of Hand and Foot Surgery, Shandong Provincial Hospital Affiliated to Shandong First Medical University, Jinan, 250021 China; 2 Department of Central Laboratory, Shandong Provincial Hospital Affiliated to Shandong University, Jinan, 250021 China; 3 Department of Reproductive Medicine, Shandong Provincial Hospital Affiliated to Shandong First Medical University, Jinan, 250021 China; 4 Department of Critical Care Medicine, Shandong University of Traditional Chinese Medicine, Jinan, 250000 China

**Keywords:** Targeted bone remodelling, Mechanisms of disease

## Abstract

The mutations of *HOXD13* gene have been involved in synpolydactyly (SPD), and the polyalanine extension mutation of *Hoxd13* gene could lead to SPD in mice. In this study, a novel missense mutation of *Hoxd13* (NM_000523: exon2: c.G917T: p.R306L) was identified in a Chinese family with SPD. The mice carrying the corresponding *Hoxd13*mutation were generated. The results showed that the homozygous mutation of *Hoxd13* also caused SPD, but heterozygous mutation did not affect limbs development, which was different from that of SPD patients. With the increasing generation, the mice with homozygous *Hoxd13* mutation presented more severe syndactyly. Western blotting showed that this mutation did not affect the protein expression of *Hoxd13*, suggesting that this mutation did not result in haploinsufficiency. Further analysis demonstrated that this homozygous *Hoxd13*mutation promoted osteoclast differentiation and bone loss, and enhanced the mRNA and protein expression of osteoclast-related genes *Rank*, *c-Fos*, and *p65*. Meanwhile, this homozygous *Hoxd13* mutation elevated the level of phosphorylated Smad5 (pSmad5). Co-immunoprecipitation verified that this mutation attenuated the interaction between pSmad5 and HOXD13, suggesting that this mutation released more pSmad5. Inhibition of pSmad5 reduced the expression of Rank, c-Fos, and p65 despite in the mutation group. In addition, inhibition of pSmad5 repressed the osteoclast differentiation. ChIP assay confirmed that p65 and c-Fos could bind to the promoter of *Rank*. These results suggested that this novel *Hoxd13* mutation promoted osteoclast differentiation by regulating Smad5/p65/c-Fos/Rank axis, which might provide a new insight into SPD development.

## Introduction

Congenital syndactyly contains simple syndactyly and complex syndactyly. Simple syndactyly, also known as cutaneous syndactyly, refers to only skin and soft tissue. Complex syndactyly, also named osseous syndactyly, refers to bone fusion or neurovascular connection between two or more fingers [1]. Osseous syndactyly, especially synpolydactyly (SPD), often involves both hands and feet. Severe limb malformation not only affects the appearance of the hand, but also seriously disturbs the function of the hand, which greatly reduces the quality of life and work ability of patients.

The mutations of homeobox *D13* (*HOXD13*) gene have been widely involved in SPD malformation [2–4]. *HOXD13* is a member of the homobox transcription factor family and plays an important role in embryonic development [5]. Polyalanine extensions in the *Hoxd13* gene have been demonstrated to induce SPD phenotype in mice by decreasing retinoic acid synthesis [6, 7]. Missense mutation of *HOXD13* (G220V) gene also causes SPD phenotype, and impairs the transcriptional activity of *HOXD13* [8]. An N-terminal G11A mutation in *HOXD13* leads to SPD phenotype by interfering with Gli3R function [9]. In addition to classical SPD caused by N-terminal polyalanine extensions or truncation, mutations of homeobox domain in exon 2 could cause atypical SPD [10–15]. Three frameshift mutations in the *HOXD13* gene result in a truncated protein with homeodomain deficiency, which prevents the HOXD13 protein from binding to the promoters of target genes, revealing the haploinsufficiency of *HOXD13* [10, 16, 17]. Furthermore, a variety of mutations including S308C, I314L, and Q317R have been associated with SPD [18–20], while the molecular mechanism of which remains largely unclear.

In the present study, a novel missense mutation of *HOXD13* (NM_000523: exon2: c.G917T: p.R306L) was identified in the fifteen individuals with SPD, and this mutation did not cause haploinsufficiency of *HOXD13* However, this homozygous *Hoxd13* mutation promoted osteoclast differentiation, and enhanced the expression of osteoclast-related genes *Rank*, *c-Fos*, and phosphorylated *p65* (*p-p65*). This mutation attenuated the interaction between HOXD13 and pSmad5, which was responsible for activation of c-Fos and p65 as well as the expression of Rank. These results provided a novel insight into the SPD development.

## Results

### A novel missense mutation of *HOXD13* was identified in a Chinese family with SPD

**Fig. 1:**
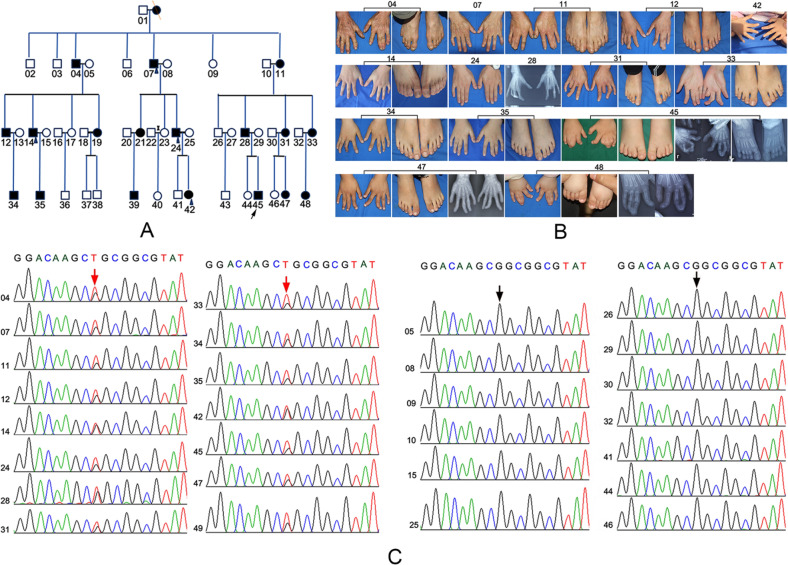
A novel missense mutation of *Hoxd13* was identified in a Chinese family with synpolydactyly (SPD). **A** The pedigree of a four-generation Chinese family with SPD. Arrow represented the proband. Circles and squares represented female and male, respectively. Blank and black represented the unaffected and affected members, respectively. **B** The clinical characteristics of affected members. **C** Whole-exon sequencing and Sanger sequencing were performed to identify the mutation of *Hoxd13* in the affected members.

The pedigree of the Chinese family with SPD was shown in Fig. 1A. In this family, this phenotype affected four successive generations composed of 48 members, among of which contained 19 affected members. The proband (No. 45) was a 1.5-year-old boy with SPD. SPD of the middle and ring fingers was observed in both hands. The distal phalanx at the end of the middle and ring fingers was skeletally connected, and there was an excess phalanx between the two fingers (Fig. 1B). The middle and end phalanges of the index and little fingers were deformed with deflection (Fig. 1B). SPD of the second and third toes was observed in both feet. In this study, 28 members were enrolled, including 15 affected members and 13 unaffected family members. Whole-exome sequencing was performed to identify the DNA mutations in affected members compared to the healthy controls. The results showed that a novel missense mutation in *HOXD13* (nm_000523: exon2: c.g917t: p.r306l) was observed in all 15 affected members, but not in all 13 unaffected members (Fig. 1C), which were confirmed by Sanger sequencing.

### The *Hoxd13* mutation caused the SPD phenotype in mice

**Fig. 2:**
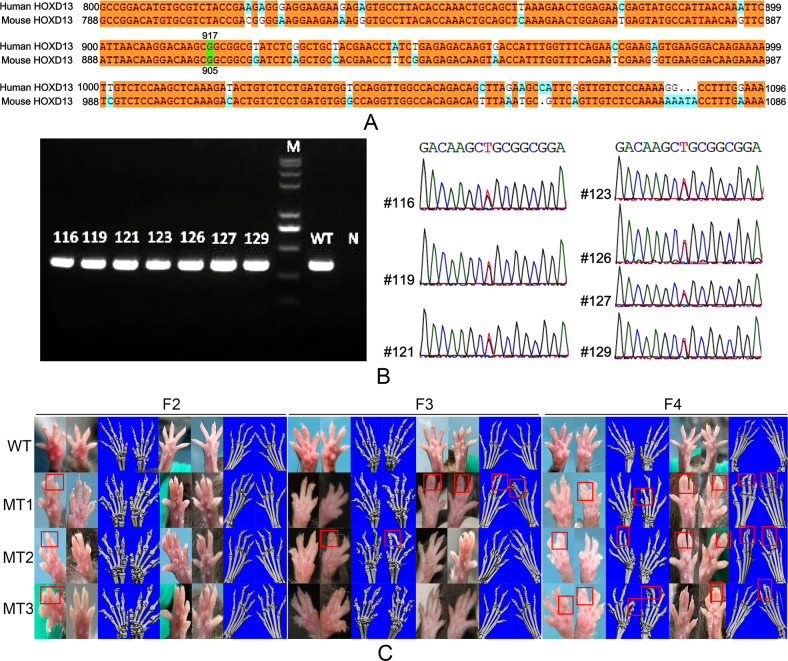
The *Hoxd13* mutation caused the SPD phenotypes in mice. **A** The sequence alignment of *Hoxd13* from human and mouse. **B** Agarose gel electrophoresis and Sanger sequencing were performed to analyze the genotypes of mice. **C** The images of mice limbs were captured by micro-CT. All experiments were repeated three times.

We next constructed the transgenic mice carrying *Hoxd13* mutation (G905T) according to the sequence alignment (Fig. 2A). All the F1 generation mice (No. 116, 119, 121, 123, 126, 127, and 129) were heterozygous by PCR and sequencing (Fig. 2B). Interestingly, in F2 generation mice, only homozygous *Hoxd13* mutation caused the obvious SPD phenotype, while did not lead to the fusion of bones visualized by micro CT (Fig. 2C). As increasing generations, homozygous *Hoxd13* mutation significantly caused skeletal syndactylia (Fig. 2C), similar to the characteristics of patients with SPD.

### The *Hoxd13* mutation promoted osteoclast differentiation and bone loss

**Fig. 3:**
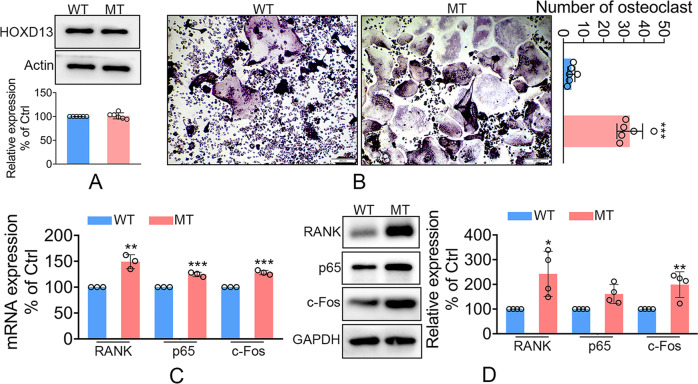
The *Hoxd13* mutation promoted the osteoclast differentiation. **A** The effect of *Hoxd13* mutation on the expression of HOXD13 protein. **B** The effect of *Hoxd13* mutation on osteoclast differentiation analyzed using TRAP staining. ***p < 0.001. **C** The effect of *Hoxd13* mutation on the mRNA expression of osteoclast-associated genes. **p < 0.01; ***p < 0.001. **D** The effect of *Hoxd13* mutation on the expression of osteoclast-associated proteins. *p < 0.05; **p < 0.01. All experiments were repeated at least three times.

**Fig. 4:**
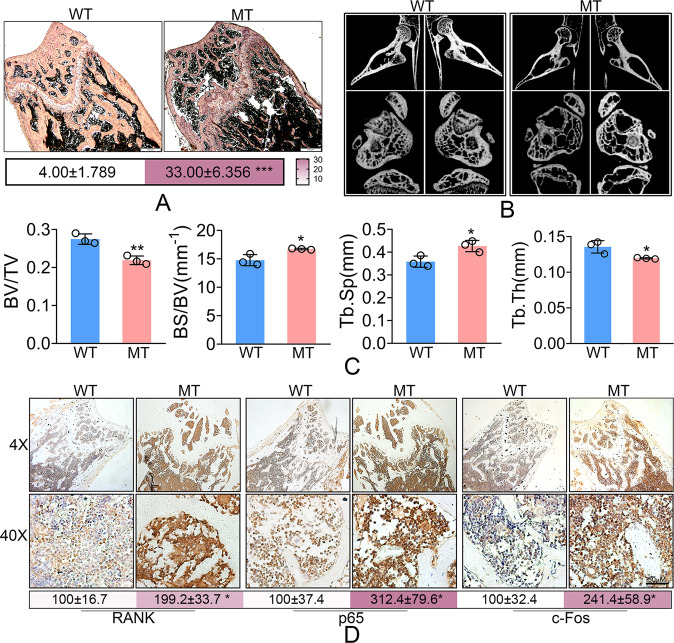
The *Hoxd13* mutation caused bone loss in vivo. **A** The effect of *Hoxd13* mutation on the osteoclast differentiation in bone tissues. **B** In vivo imaging of the tibia and femur from mice carrying wild and mutant *Hoxd13* by micro-CT. **C** The BV/TV, BS/BV, Tb.Sp, and Tb.Th obtained using micro-CT. *p < 0.05; **p < 0.01. **D** Immunohistochemistry assay was used to determine the expression of Rank, p65, and c-Fos in bone tissues. *p < 0.05. All experiments were repeated three times.

We further examined the differential expression of *Hoxd13* between the wild and mutant *Hoxd13* mice. The results showed that the *Hoxd13* mutation did not affected the protein expression of *Hoxd13* (Fig. 3A), suggesting that the *Hoxd13* mutation did not cause the haploinsufficiency. Bone marrow monocytes (BMMs) were isolated from wild and mutant *Hoxd13* mice, and were exposed to M-CSF and RANKL for 5 days. As shown in Fig. 3B, the *Hoxd13* mutation significantly promoted the osteoclast differentiation (Fig. 3B). To confirm this result, we tested the expression of osteoclast-associated proteins, including Rank, p65, and c-Fos. The results indicated that the *Hoxd13* mutation elevated both mRNA and protein expression of *Rank*, *p65*, and *c-Fos* (Fig. 3C, D). The femur and tibia from wild and mutant *Hoxd13* mice were analyzed by TRAP staining. The results showed that the *Hoxd13* mutation notably increased the osteoclast differentiation and rarefaction of bone (Fig. 4A). The in vivo imaging of femur and tibia from mice carrying wild and mutant *Hoxd13* was performed using micro-CT. The results demonstrated that this *Hoxd13* mutation caused bone loss (Fig. 4B). The BV/TV and Tb.Th were decreased in the *Hoxd13* mutant mice compared to wild mice, while the BS/BV and Tb.Sp were increased in *Hoxd13* mutant mice (Fig. 4C). In addition, the expression of Rank, p65, and c-Fos were upregulated in the bone tissues from *Hoxd13* mutant mice using immunohistochemistry analysis (Fig. 4D). These results suggested that the *Hoxd13* mutation promoted osteoclast differentiation and bone loss by elevating the expression of Rank, c-Fos, and p65.

### The *Hoxd13* mutation increased the expression of Rank, c-Fos, and phosphorylated p65 by releasing pSmad5

**Fig. 5:**
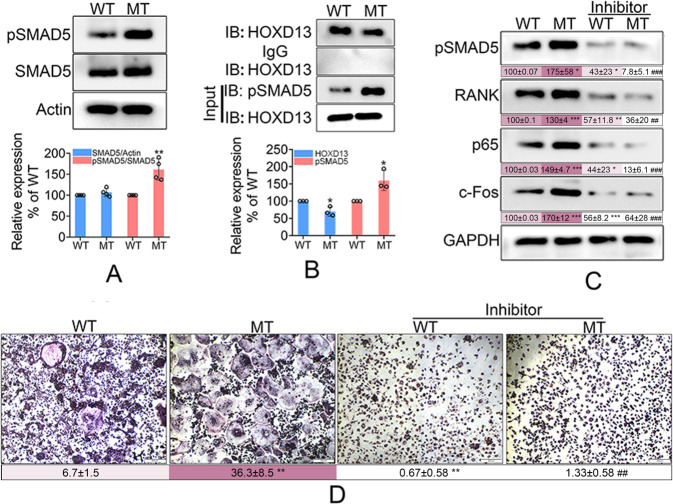
The *Hoxd13* mutation increased the expression of Rank, c-Fos, and phosphorylated p65 by releasing pSmad5. **A** The effect of *Hoxd13* mutation on the expression and phosphorylation of Smad5 (pSmad5). **p < 0.01. **B** The effect of *Hoxd13* mutation on the interaction between HOXD13 and pSmad5. *p < 0.05. **C** The bone marrow monocytes from wild and mutant mice were treated with 1200 nM dorsomorphin, and were then exposed to M-CSF and RANKL for 5 days. Western blot was performed to determine the expression of pSmad5, Rank, p65, and c-Fos. *p < 0.05; **p < 0.01; ***p < 0.001; ^##^*p* < 0.01 vs MT groups; ^###^*p* < 0.001 vs MT groups. **D** TRAP staining was performed to determine the osteoclast differentiation. **p < 0.01; ^##^*p* < 0.01 vs MT groups. All experiments were repeated three times.

It is necessary to explore the regulatory mechanism of *Hoxd13* in the expression of Rank, c-Fos, and p65. The results demonstrated that the *Hoxd13* mutation did not affect the expression of total Smad5, but promoted the phosphorylation of Smad5 (pSmad5) (Fig. 5A). Co-Immunoprecipitation assay confirmed that the *Hoxd13* mutation reduced the interaction between HOXD13 and pSmad5 (Fig. 5B), suggesting that the *Hoxd13* mutation increased more free pSmad5. Inhibition of Smad5 phosphorylation simultaneously restrained the expression of Rank, c-Fos, and phosphorylated p65 (p-p65) (Fig. 5C), indicating that pSmad5 participated in the induction of Rank, c-Fos, and p-p65. TRAP staining verified that pSmad5 inhibition blocked the *Hoxd13* mutation-induced osteoclast differentiation (Fig. 5D). These results suggested that the *Hoxd13* mutation increased the expression of Rank, c-Fos, and p-p65 by releasing pSmad5.

### The *Hoxd13* mutation might promote the transcription of *Rank* by regulating the expression of p65 and c-Fos

**Fig. 6:**
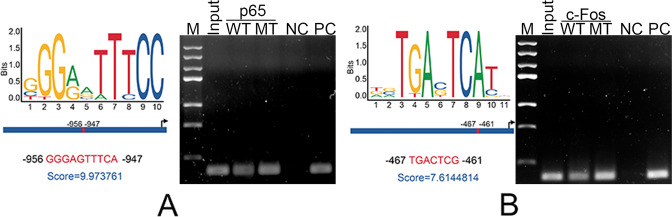
The *Hoxd13* mutation regulated the transcription of *Rank* via p65 and c-Fos. **A** The probable binding site of p65 in the promoter of *Rank* was predicted by JASPAR, and was confirmed by ChIP assay. **B** The probable binding site of c-Fos in the promoter of *Rank* was predicted by JASPAR, and was confirmed by ChIP assay. All experiments were repeated three times.

Considering that c-Fos and p65 serve as transcriptional factors in the multiple biological processes, we predicted the likely binding sites of c-Fos and p65 in the promoter of *Rank* using JASPAR. The functions of c-Fos and p65 in the transcription of *Rank* were determined by ChIP assay. The results revealed that p65 and c-Fos could bind to the promoter of *Rank*, which was enhanced by the *Hoxd13* mutation (Fig. 6A, B). These results suggested that the *Hoxd13* mutation might promote the transcription of *Rank* by regulating the expression of p65 and c-Fos.

## Discussion

Although the roles of *HOXD13* mutations in SPD are well known, the functional mechanism of *HOXD13* mutations remains largely unknown. The *HOXD13* gene consists of two exons. Exon 1 contains 45 bp trinucleotide repeats encoding a 15-residue polyalanine expansion at the N-terminal of *HOXD13* [21]. Exon 2 is a 180 bp homeobox domain, which is a highly conserved DNA-binding domain at the C terminus of *HOXD13* [21]. *HOXD13*, like other *HOX* proteins, regulates the transcription of target genes by binding to the their promoters through the homeodomain [2, 22]. So far, all mutations identified are localized to the N-terminal polyalanine expansions or the C-terminal homeodomain of *HOXD13*, resulting in classical or nonclassical SPD, respectively [12, 13, 23, 24]. In this study, we identified a new *HOXD13* mutation in a Chinese family with SPD. This *Hoxd13* mutation did not lead to haploinsufficiency, but promoted osteoclast differentiation in mice. Bone remodeling and repair are exactly regulated by interaction between osteoblasts and osteoclasts during embryonic and postnatal development [25–27]. Osteoblasts secrete bone matrix proteins (BMPs) and osteoclasts control bone resorption and clearance [28]. Most bone diseases are caused by the imbalance between osteoblasts and osteoclasts [29]. However, the association between osteoclast and SPD has never been explored.

To confirm the fact that the *HOXD13* mutation triggered osteoclast differentiation, we further tested the expression of osteoclast-associated proteins. The results indicated that this *Hoxd13* mutation significantly elevated the expression of Rank, c-Fos, and p65. Rank is mainly expressed on the membrane of osteoclast precursors, and acts as the receptor of Rankl secreted by osteoblasts [30, 31]. The interaction between Rank and Rankl activates TNF receptor-associated factors (TRAF), including TRAF2, TRAF5, and TRAF6 [32], triggering the downstream signals, such as NFATC1, c-Fos, and NFκB [33], which is consistent with our results. Meantime, we evidenced that this *Hoxd13* mutation also caused the osteoclast differentiation and rarefaction of bone. These results confirmed that this *Hoxd13* mutation promoted osteoclast differentiation. Previous studies have demonstrated that SPD may be related to abnormal joint formation [34] or abnormal chondrocyte differentiation and proliferation [7]. Our results linked SPD to the osteoclast differentiation. Furthermore, *Hoxd13* mutation has been involved the cell polarity in the perichondrium [35]. The *Nup98*-*Hoxd13* (*NHD13*) transgenic mice display increasing osteoblasts, endothelial cells, dysfunctional mesenchymal cells, and decreasing megakaryocytes [36, 37]. Combined our results, these findings suggested key roles of HOXD13 in the most cell types in the bone marrow microenvironment.

However, the molecular mechanism of the *HOXD13* mutation in osteoclast differentiation remained largely unknown. *HOXD13* mutations in the homeobox domain have dominant-negative effects through interactions with other proteins [38]. HOX proteins have been demonstrated to interact with the MH2 domain of Smad proteins [39]. HOXD13 could bind to BMP proteins and TGF-β-mediated Smad protein including Smad1 and Smad2, but not Smad4 [39]. In this study, we found that this *Hoxd13* mutation promoted the phosphorylation of Smad5. Further investigation verified that this *Hoxd13* mutation attenuated the interaction between HOXD13 and phosphorylated Smad5 (pSmad5), suggesting that this *Hoxd13* mutation released pSmad5, which might be responsible for the expression of NFκB (p65) and c-Fos. Dorsomorphin, a small-molecule inhibitor of Smad5 signaling [40, 41], simultaneously reduced the levels of pSmad5, Rank, p65, and c-Fos, indicating that increasing pSmad5 contributed to the expression of Rank, p65, and c-Fos. Considering that this *Hoxd13* mutation promoted the expression of pSmad5 and attenuated the interaction between HOXD13 and pSmad5, it was likely that elevating free pSmad5 enhanced the expression of p65 and c-Fos in the osteoclasts, which was consistent with previous findings.

Finally, how did this *Hoxd13* mutation regulate the expression of Rank? Since this *Hoxd13* mutation promoted the expression of p65 and c-Fos, we analyzed the probable binding sites of p65 and c-Fos in the promoter of *Rank*. The results showed that both p65 and c-Fos could bind to the promoter of *Rank*, which was enhanced by this *Hoxd13* mutation. Combined the simultaneous increase in the levels of p65, c-Fos, and Rank in *Hoxd13* mutated osteoclasts, these results suggested that p65 and c-Fos might positively regulate the transcription of *Rank*, and HOXD13 regulated RANK expression via p65 and c-Fos. Although p65 and c-Fos are closely associated with the osteoclast differentiation [42, 43], they have never been demonstrated to regulate the transcription of *Rank*.

**Fig. 7:**
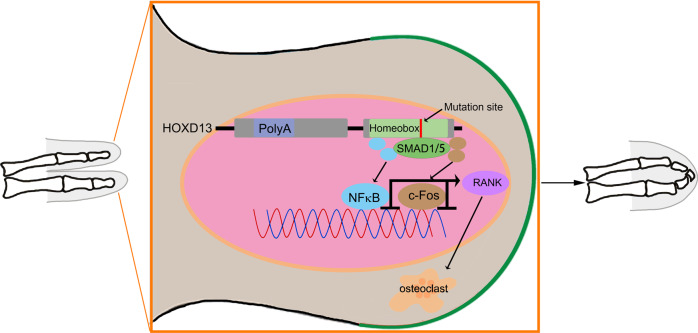
The *Hoxd13* mutation promoted osteoclast differentiation by regulating pSmad5/p65/c-Fos/Rank axis. Schematic diagram described the possible functional mechanism of the *Hoxd13* mutation in the osteoclast differentiation.

In summary, we identified a novel missense mutation in *HOXD13* in a Chinese family with SPD, and the similar phenotypes were observed in mice carrying the corresponding *Hoxd13* mutation. This *Hoxd13* mutation did not cause a haploinsufficiency, but promoted the osteoclast differentiation. Further investigation demonstrated that this *Hoxd13* mutation increased the phosphorylation of Smad5 (pSmad5), and attenuated the interaction between HOXD13 and pSmad5, suggesting that this *Hoxd13* mutation released pSmad5, which elevated the expression of p65 and c-Fos. p65 and c-Fos bound to the promoter of *Rank* to initiate its transcription (Fig. 7).

## Materials and methods

### Materials

The antibodies were used as follows: anti-HOXD13 (pAb,18736-1-AP), anti-GAPDH (pAb, 10494-1-AP), anti-beta-actin (pAb, 20536-1-AP), anti-SMAD5 (pAb, 12167-1-AP), anti-NF-κB p65 (pAb, 10745-1-AP) were purchased from Proteintech (Proteintech Group Inc, Rosement, USA); anti-anti-phospho-SMAD5 (mAb, #13820) was purchased from Cell Signaling Technology (Beverly, MA); anti-c-Fos (pAb, A0236), anti-TNFRSF11A (pAb, A12997) were purchased from ABclonal (Wuhan, Hunan, China); anti-c-Fos (TA0132S) was purchased from ABMART (Shanghai, China); anti-IgG (bs-0295PC) was purchased from BIOSS (Beijing, China). M-CSF (416-ML-010/CF) and RANKL (462-TEC-010/CF) were purchased from R&D System (MN, USA). The chip kit (ab500) was purchased from Abcam (Cambridge, MA). The Co-IP kit (26149) was purchased from Thermo Fisher Scientific (MA, USA). The TRAP stain kit (387A) was purchased from Sigma-Aldrich (MO, USA). Minimum Essential medium (α-MEM) and fetal bovine serum (FBS) were purchased from Gibco Life Technologies (Grand Island, NY, USA).

### Cell culture

Cells were derived from the bone marrow and cultured in a humidified incubator at 37 °C containing 5%CO_2_. The complete medium was α-MEM medium containing 10% FBS and 1% penicillin-streptomycin.

### Construction of mutant mice

CRISPR/Cas9 technology was used to modify the target site through the principle of homologous recombination. The specific process was as follows: gRNA was designed and transcribed in vitro, and the homologous recombination vector (Donor vector) was constructed. Cas9, gRNA, and Donor vector were injected into the fertilized eggs of mice at the same time. Under the guidance of gRNA, Cas9 protein binds to the target site and causes DNA double-strand break. The Donor vector repairs the broken double-strand through homologous recombination to achieve gene knock-in at the target site. The sequence of gRNA: 5′-AGTTCATTAACAAGGACAAG-3′, and the sequence of Donor: TATGCCATTAACAAGTTCATTAACAAGGACAAGCTGCGGCGGATCTCAGCTGCCACGAACCTTTCGGAGAGACA (underlined base is the targeted mutation). Mice were maintained under specific pathogen-free conditions in the animal facility, and all experiments were approved by the Laboratory Animal Ethics Committee of Shandong Provincial Hospital.

### Isolation and differentiation of bone marrow monocyte

Eight-week C57BL/6 mice (at least three mice per group) were sacrificed and soaked in 75% ethanol for 5 min. The femur and tibia were then obtained in an ultra-clean table, and placed in the sterile PBS. Subsequently, the bone marrow cavities were rinsed using α-MEM medium until it was changed to white. The cell suspensions were gently mixed and were passed through 70-μm filters to remove redundant tissues, followed by centrifugation at 1200 rpm for 5 min. After removing the supernatant, the cells were gently resuspended using the α-MEM medium containing 10% FBS. Cells were incubated at 37 °C containing 5% CO_2_ for 24 h. The supernatants were discarded and the cells were incubated in the erythrocyte lysate for 5 min, followed by centrifugation at 1500 rpm for 5 min. The cells were gently washed twice using the α-MEM complete medium and centrifuged for 5 min. The harvested cells were incubated in the α-MEM complete medium containing 25 ng/ml M-CSF for 48 h. The medium were replaced with the α-MEM complete medium containing 25 ng/ml M-CSF and 40 ng/ml RANKL, and the cells were cultured for 7 days. The medium was changed every two days. Trap staining was performed to determine the osteoclast differentiation.

### TRAP staining

The cells were fixed with 4% paraformaldehyde for 30 min, and washed twice with PBS. The cells were then incubated in A staining solution and B staining solution for 30 min in the dark, respectively. After washing with PBS and natural drying, the cell images were captured using an olympus microscope (IX53).

### Genotype identification

The tails of 3-week mice were sheared into EP tubes, and were incubated in SNET solution (containing 1% SDS, 400 mM NaCl, 5 mM EDTA, 20 mM Tris) and 0.17 mg/ml protease K overnight at 56 °C. After being cooled to room temperature, saturated NaCl was added and mixed for 5 min, followed by centrifugation at 12,000 rpm for 15 min at 4 °C. The supernatants were obtained and mixed with 500 μl isopropyl alcohol on ice. After centrifugation at 12,000 rpm for 15 min at 4 °C, supernatant was removed, and the precipitate was mixed with 75% ethanol and washed once, followed by centrifugation at 7500 × *g* for 5 min at 4 °C. The precipitate was air-dried, and wad redissolved in the DEPC water.

PCR was performed using 100 ng DNA as the template (Forward primer, 5′-TTAGGTGTTCCAAGTATCCAGG3′; Reverse primer, 5′-TAAACTGTCTGTGGCCAACC-3′). PCR products were separated by the agarose gel electrophoresis. Wild and homozygous mice were randomized for the subsequent experiments.

### Quantitative real-time PCR analysis

The total RNA was obtained using the Trizol reagent, and was then reversely transcribed into cDNA using the HiScript II Q Rt Supermix for qPCR (+ GRNA Wiper) reagent kit. PCR was performed using cDNA as the template and the specific primers (*Gapdh*: Forward primer, 5′-TGTCTCCTGCGACTTCAACA-3′; Reverse primer, 5′-GGTGGTCCAGGGTTTCTTACT-3′. *Rank*: Forward primer, 5′-CCGCAGGAACACGGAGTG-3′; Reverse primer, 5′-CACCGTATCCTTGTTGAGCTGC-3′. *Nf-kb*: Forward primer, 5′-ATCGCCACCGGATTGAAGAG-3′; Reverse primer, 5′-CGGGG TTCAGTTGGTCCATT-3′. *c-Fos*: Forward primer, 5′-AGTTGATCTGTCTCCGCTT GG-3′; Reverse primer, 5′-AGAGCGGGAATGGTGAAGAC-3′).

### Western blot analysis

The total protein was extracted using the RIPA lysis buffer, and the concentration was determined using the BCA method. Equal amount of protein was loaded and separated by 10% SDS-PAGE electrophoresis. The protein was then transferred to PVDF membrane. The membrane was blocked for 2 h with 5% nonfat milk at room temperature. After washing using TBST, the membrane was incubated with the corresponding primary antibody at 4 °C overnight. After washing using TBST, the membrane was incubated with the HRP-coupled secondary antibody at room temperature for 1 h. The protein bands were visualized using the enhanced chemiluminescence method.

### Microcomputed tomography (μCT)

After inhalation anesthesia using the isoflurane, the limbs of 8-week-old mice (three mice per group) were fixed on the CT scanner, and the toes were fully spread. The upper and lower toes were scanned completely, and the regional composition was constructed into a 3D map by a computer to observe the bone condition of the mice.

### Co-Immunoprecipitation

The osteoclasts derived from *Hoxd13* wild or mutant bone marrow monocytes were harvested and incubated in the IP lysis buffer for 5 min on ice. After centrifugation at 13,000 × *g* for 10 min, the supernatants were obtained for the subsequent co-immunoprecipitation (Co-IP) assay. The Co-IP assay was performed using the Co-IP kit (Thermo Scientific Pierce) according to the manufacturer’s instructions.

### Immunohistochemistry

*Hoxd13* wild or mutant mice at 8 weeks were sacrificed. The femur and tibia were isolated and fixed with 4% paraformaldehyde for 2 days in centrifuge tubes. After rinsed with PBS, bone tissues were transferred to new centrifuge tubes and were incubated with 10% EDTA decalcification solution at 4 °C for 1 month. The decalcification solution was changed every seven days. After rinsing with water for 10 min, the bone tissues were dehydrated, embedded, and sectioned for immunohistochemistry.

### Chromatin immunoprecipitation

Chromatin immunoprecipitation (ChIP) assay was performed using the ChIP kit (Abcam, AB500). The cells were lysed and sonicated to obtain appropriate lengths of DNA fragments, and a small part of the chromatin that had been sonicated was taken to determine the length of DNA fragments. The DNA samples were quantitatively divided into three samples, including the tested group, positive control, and negative control, and the remaining DNA samples were used as the Input groups. Three samples were incubated with the corresponding antibodies and microbeads for immunoprecipitation overnight. The DNA purification was performed to obtain the DNA fragments bound to the target antibodies. Purified DNA was used as the template to amplify target fragments (Primers for *p65*: forward, 5′-GTACCGAGAAGACATAAATCGCT-3′; Reverse, 5′-TAACAAGGAAGACTGGATTTGTCT-3′. Primers for *c-Fos*: forward, 5′-GCTATGAGTGTTACAGAGGGGG-3′; Reverse, 5′-TACTTCCCCTCATTCTGGCCG-3′), and the amplified DNA fragments were analyzed by the agarose gel electrophoresis.

### Statistical analysis

GraphPad Prism 8.0 was used to carry out statistical analysis. All data represented the mean ± SD of at least three independent experiments. Comparison between two groups was performed by the Student’s t-test. *P* < 0.05 was considered statistically significant.

## Supplementary information

Reproducibility checklist

supplementary meterials

## Data Availability

All data used or analyzed during this study are included in this published article.
