# Supplementary material for: A novel Hoxd13 mutation causes synpolydactyly and promotes osteoclast differentiation by regulating pSmad5/p65/c-Fos/Rank axis
Source: Cell Death Dis. 2023 Feb 20;14(2):145. doi: 10.1038/s41419-023-05681-8 (PMC9941469; doi:10.1038/s41419-023-05681-8)
Supplement: Supplementary file 2 — supplementary meterials [file 41419_2023_5681_MOESM2_ESM.docx]

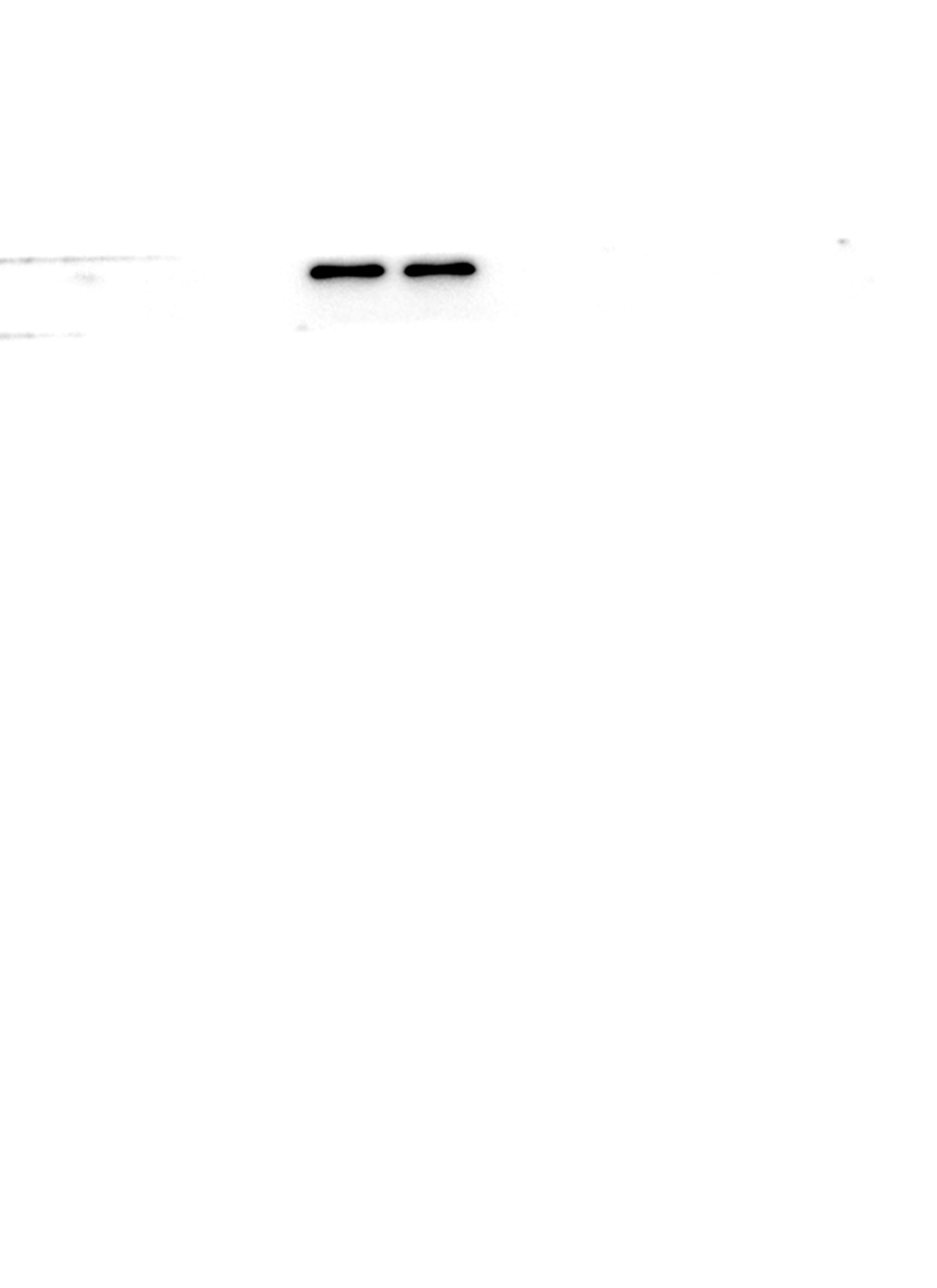


Fig.3A Western blot analysis of HOXD13


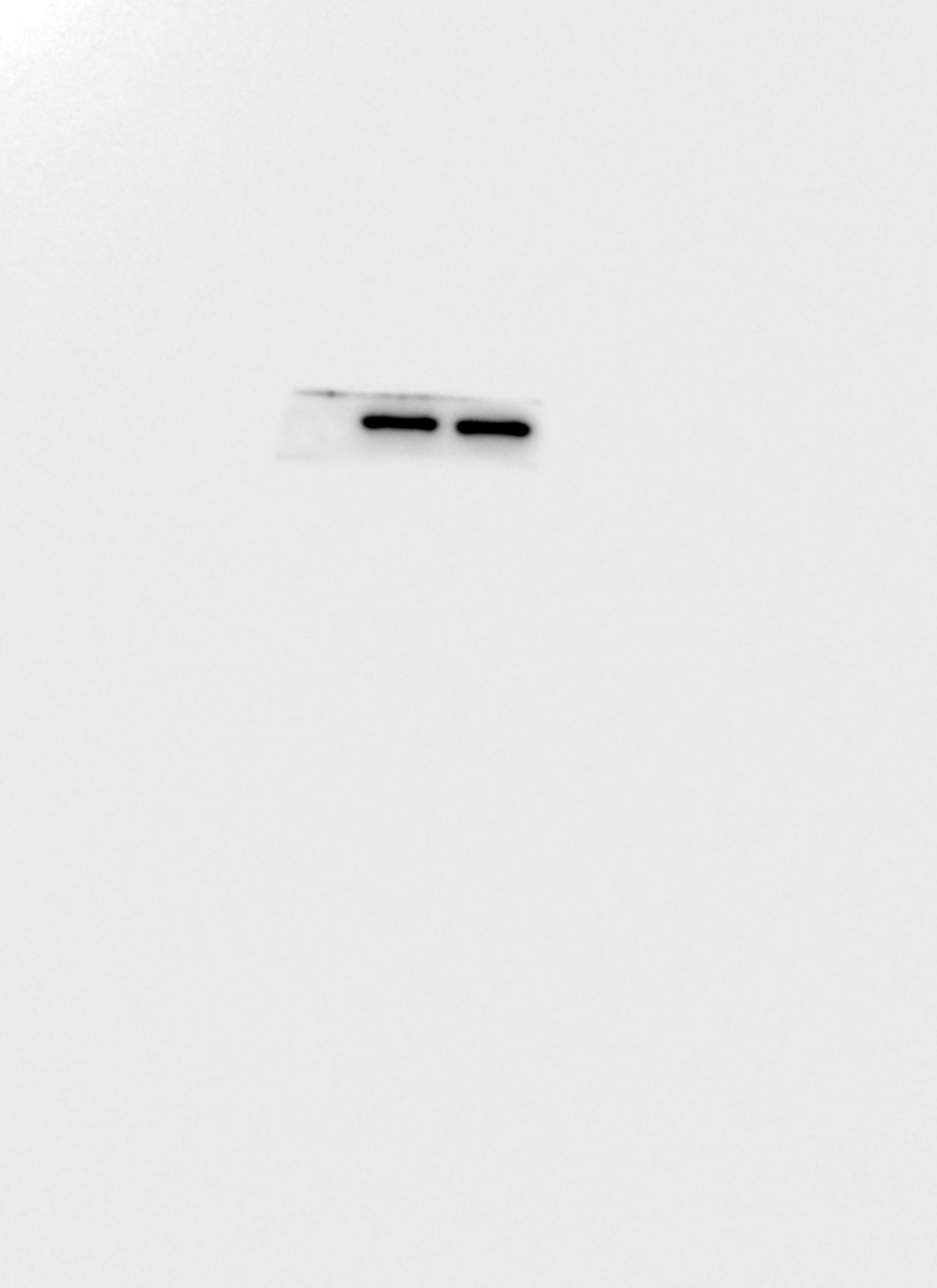


Fig.3A Western blot analysis of Actin


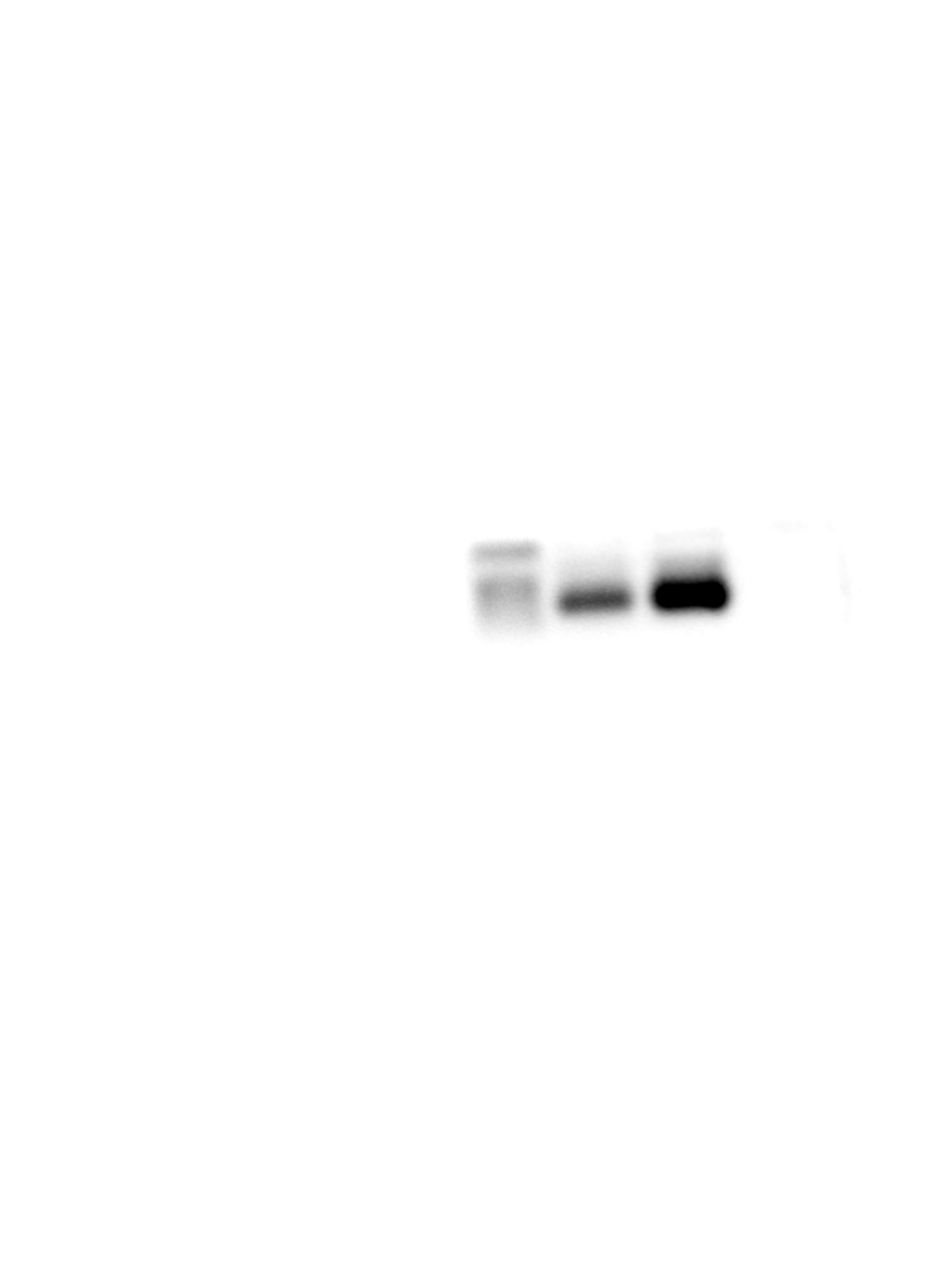


Fig.3D Western blot analysis of RANK


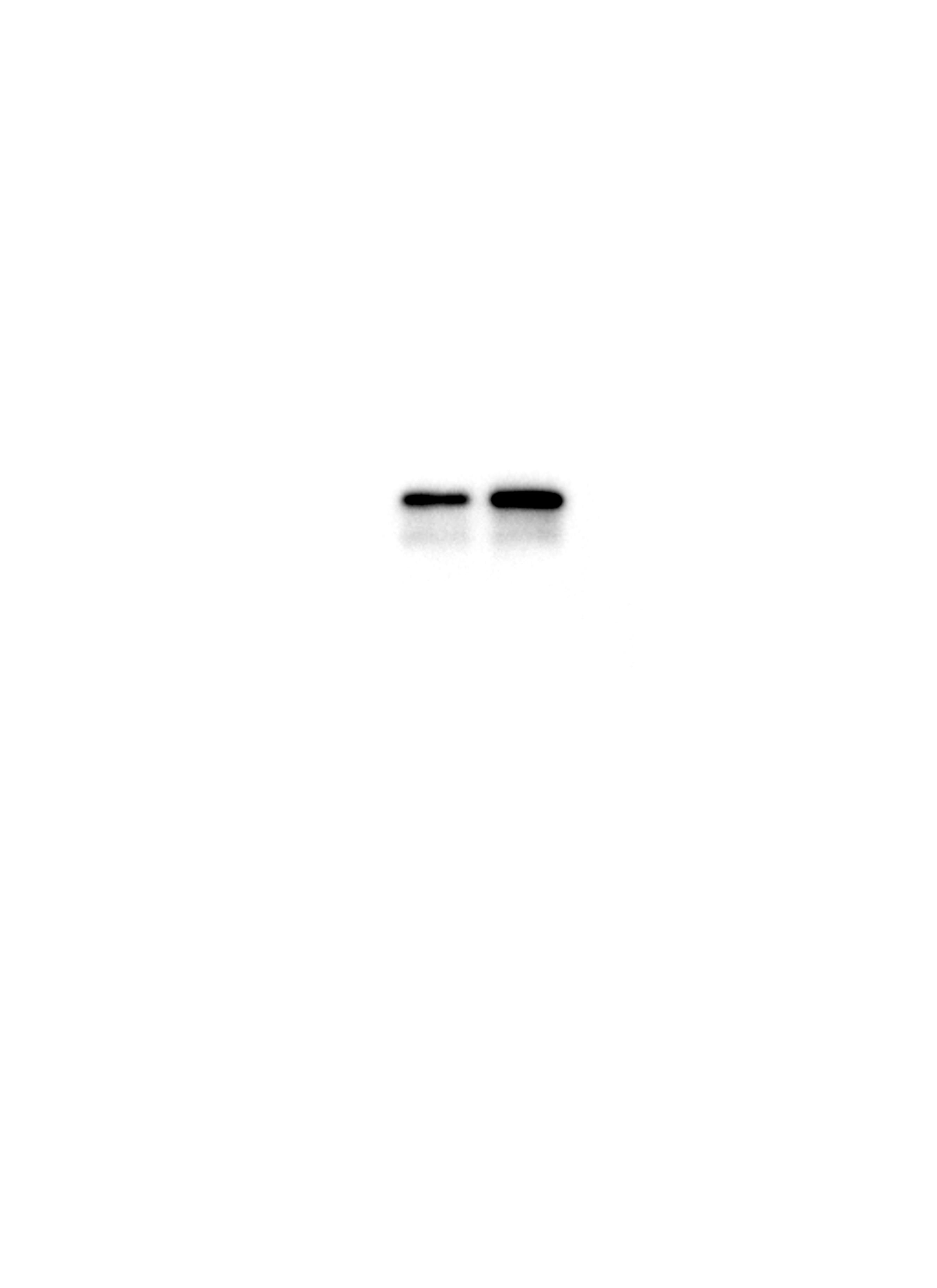


Fig.3D Western blot analysis of p65


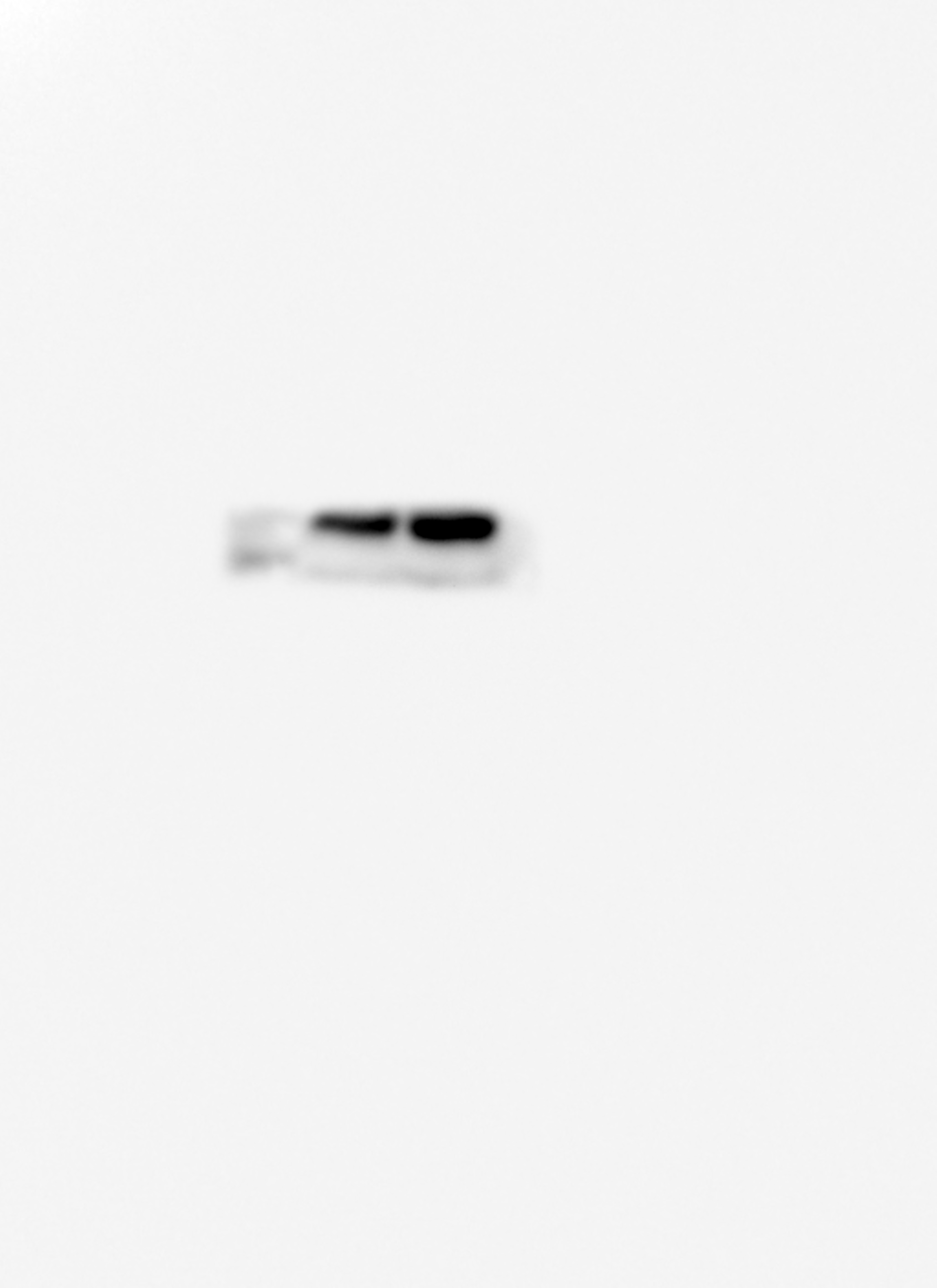


Fig.3D Western blot analysis of c-Fos


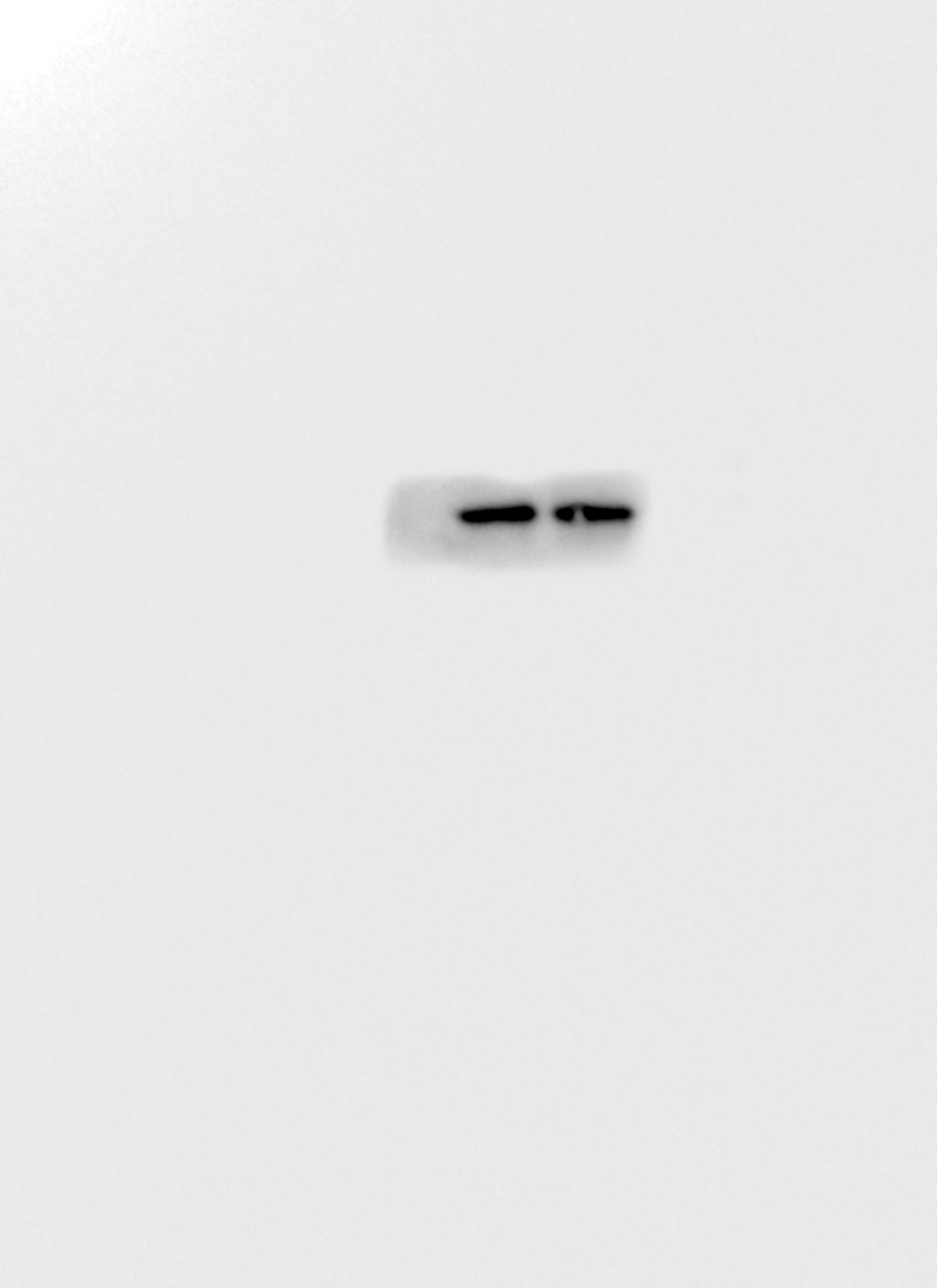


Fig.3D Western blot analysis of GAPDH


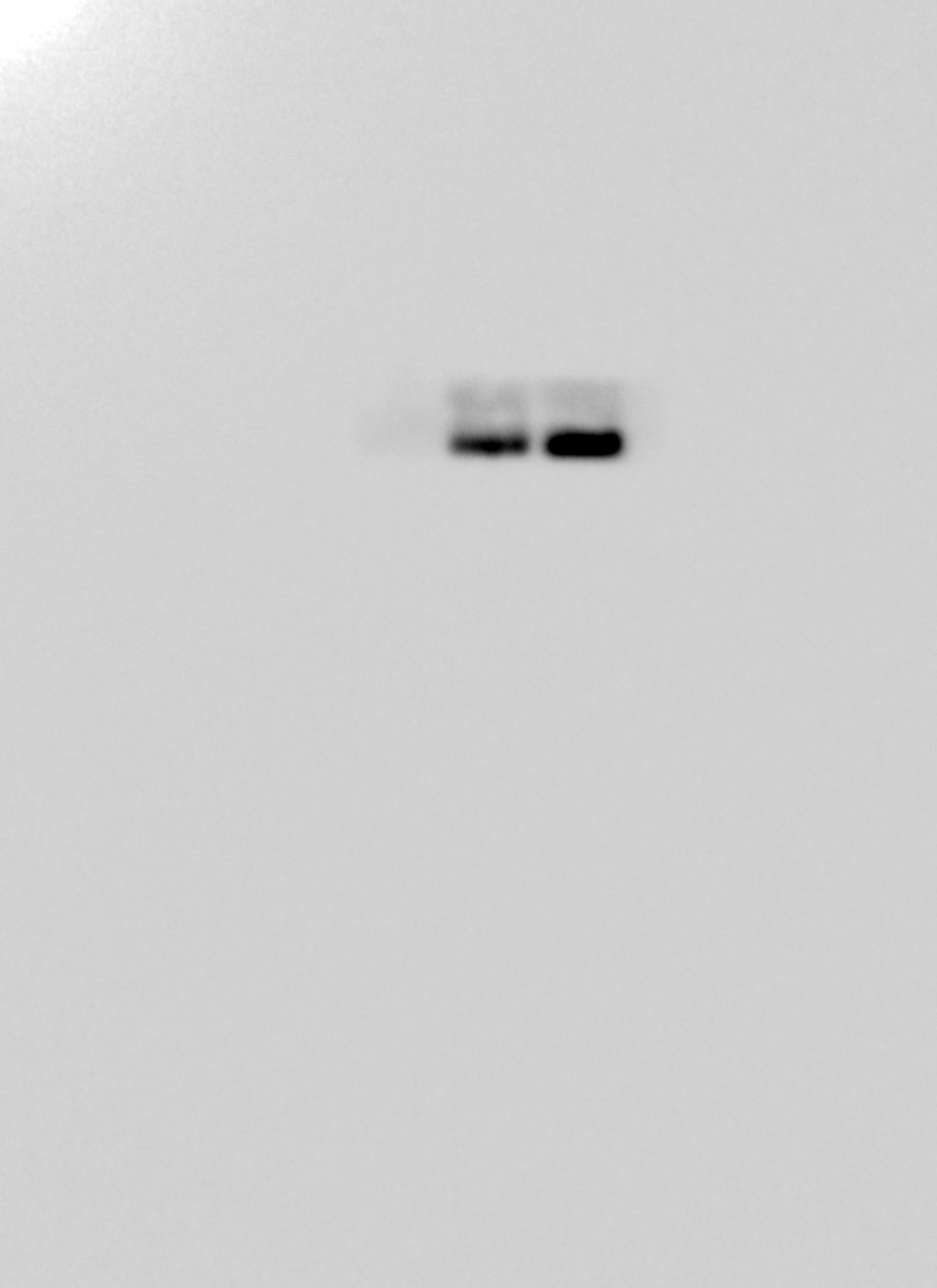


Fig.5A Western blot analysis of pSMAD5


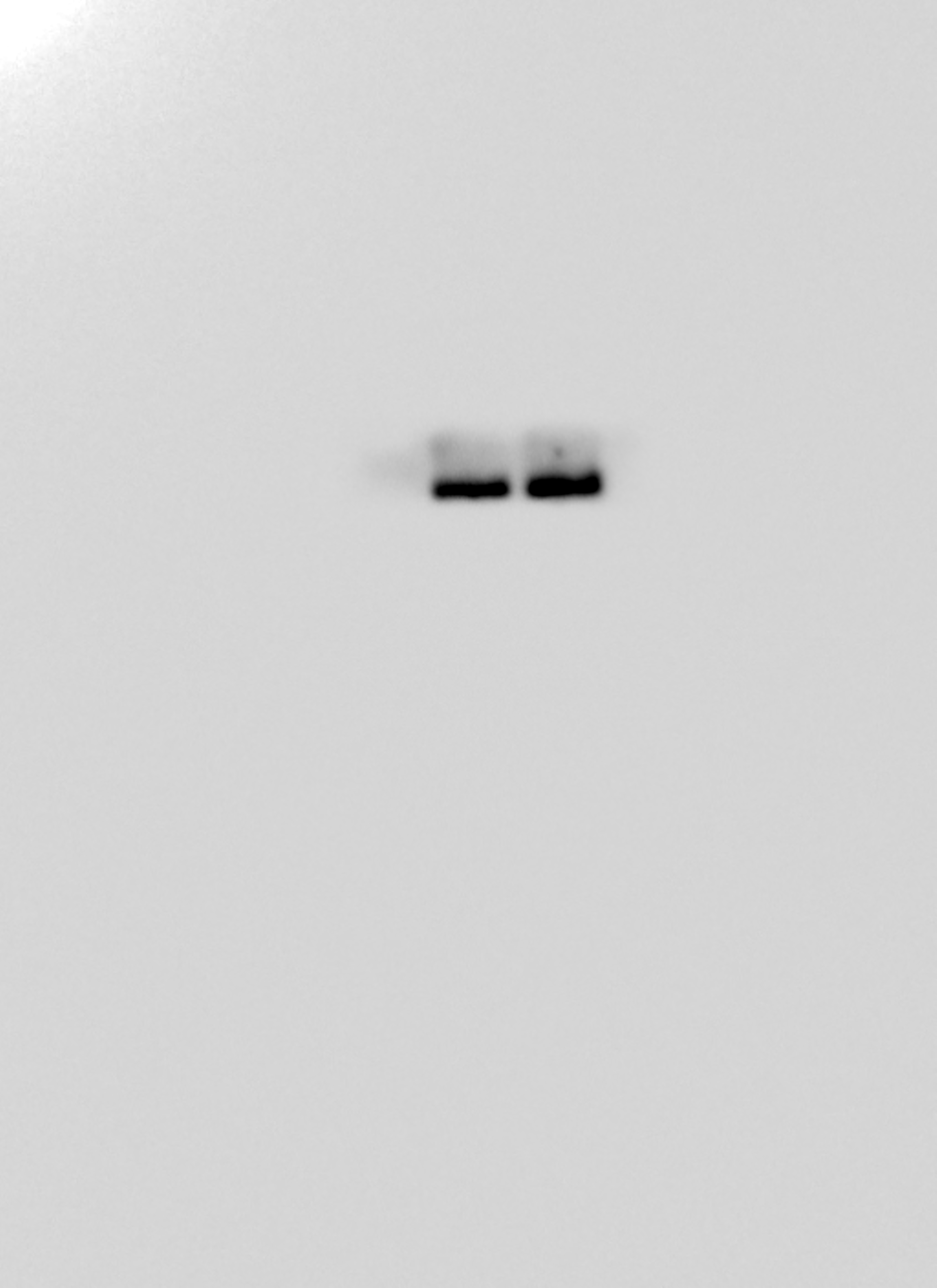


Fig.5A Western blot analysis of SMAD5


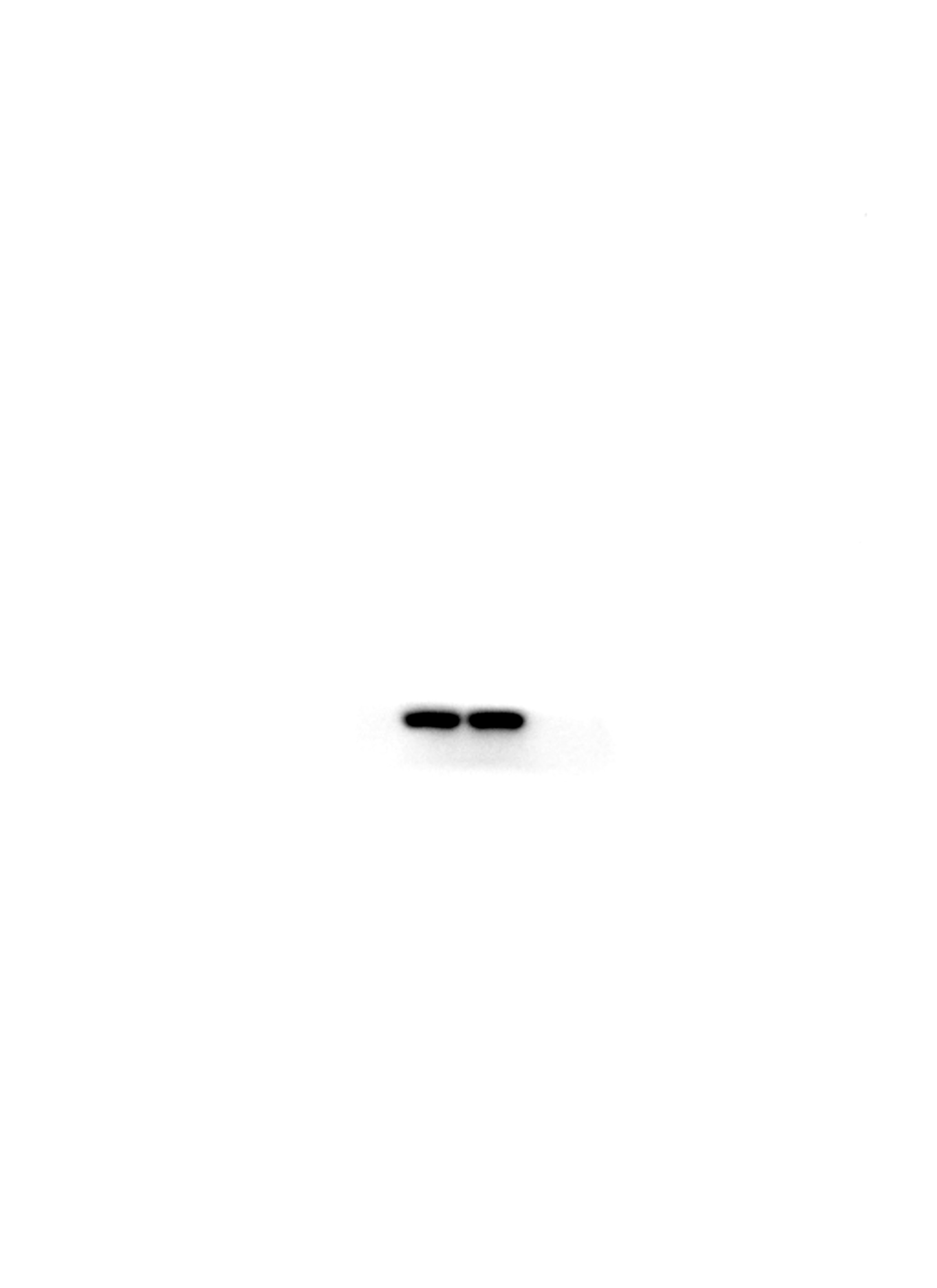


Fig.5A Western blot analysis of Actin


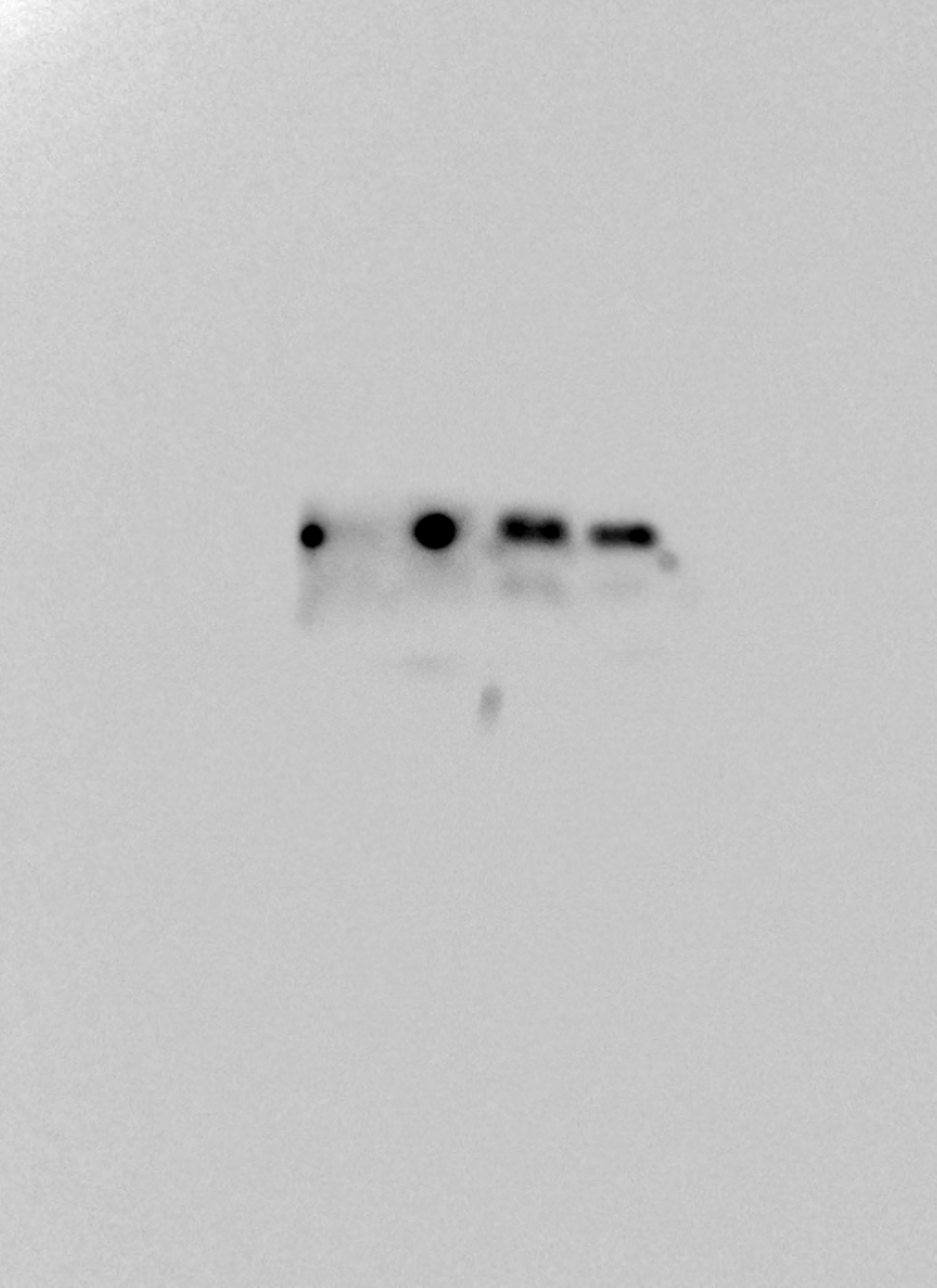


Fig.5B Western blot analysis of HOXD13 binding to pSMAD5


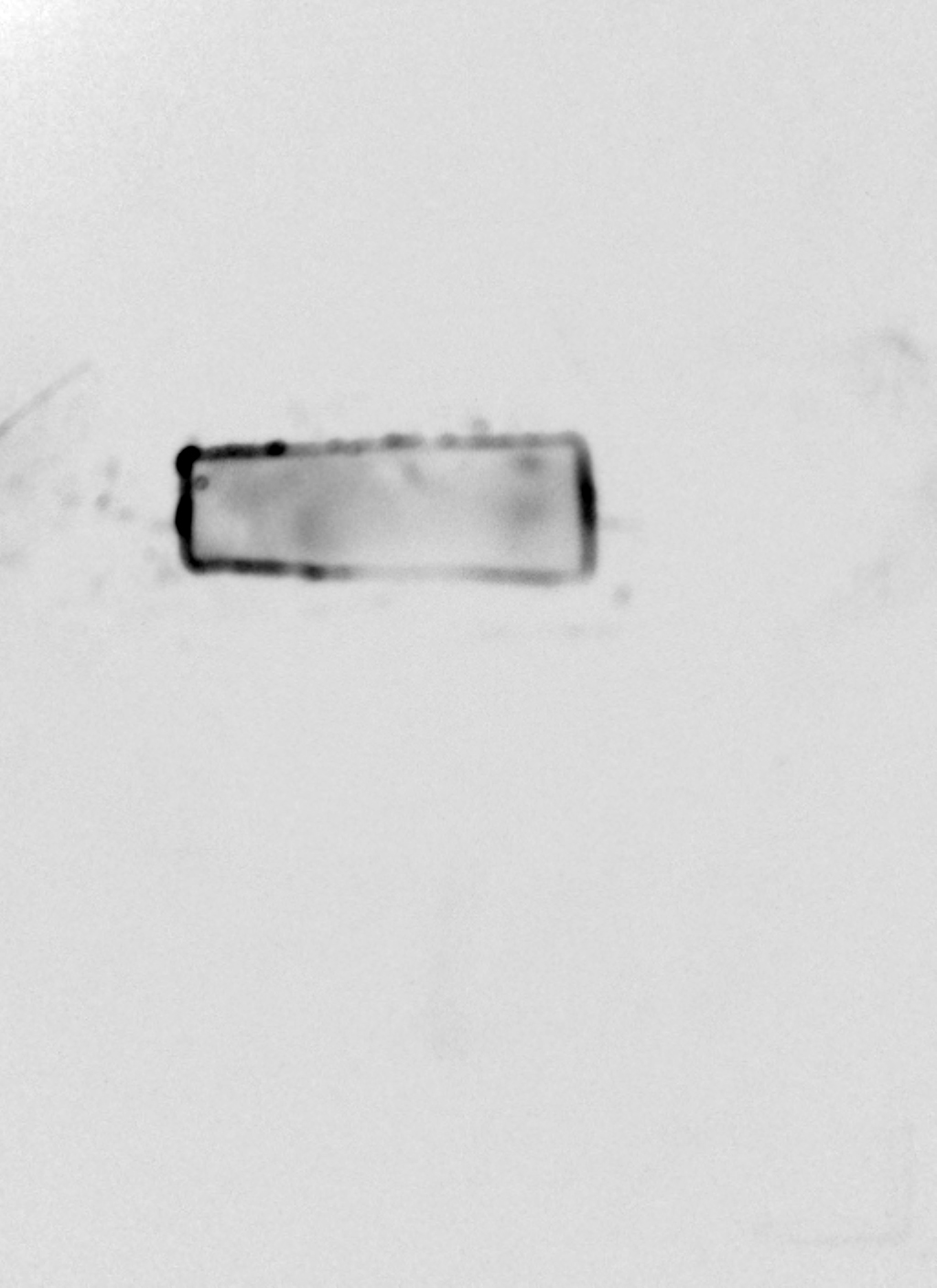


Fig.5B Western blot analysis of HOXD13 binding to IgG


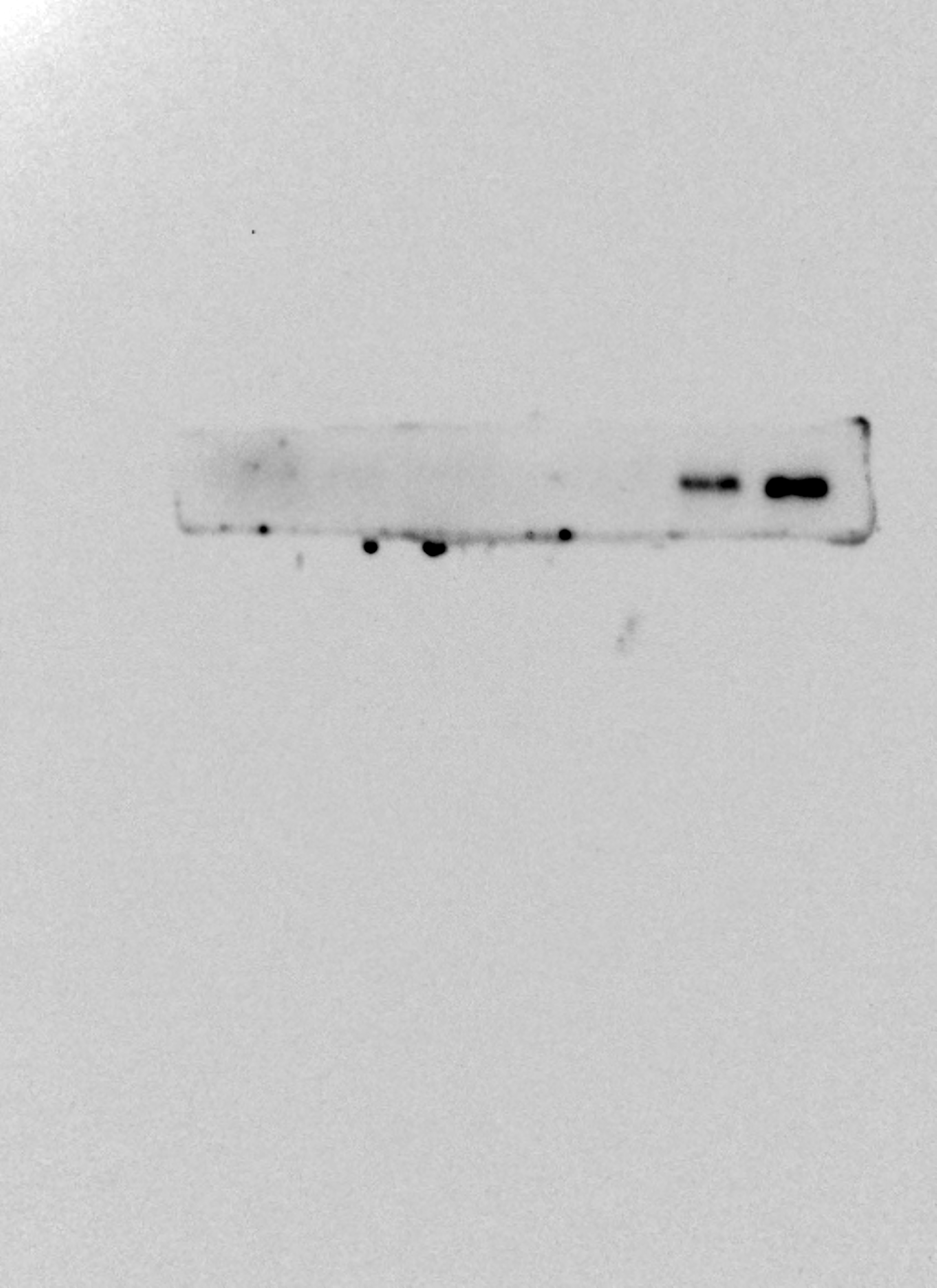


Fig.5B Western blot analysis of pSMAD5-input


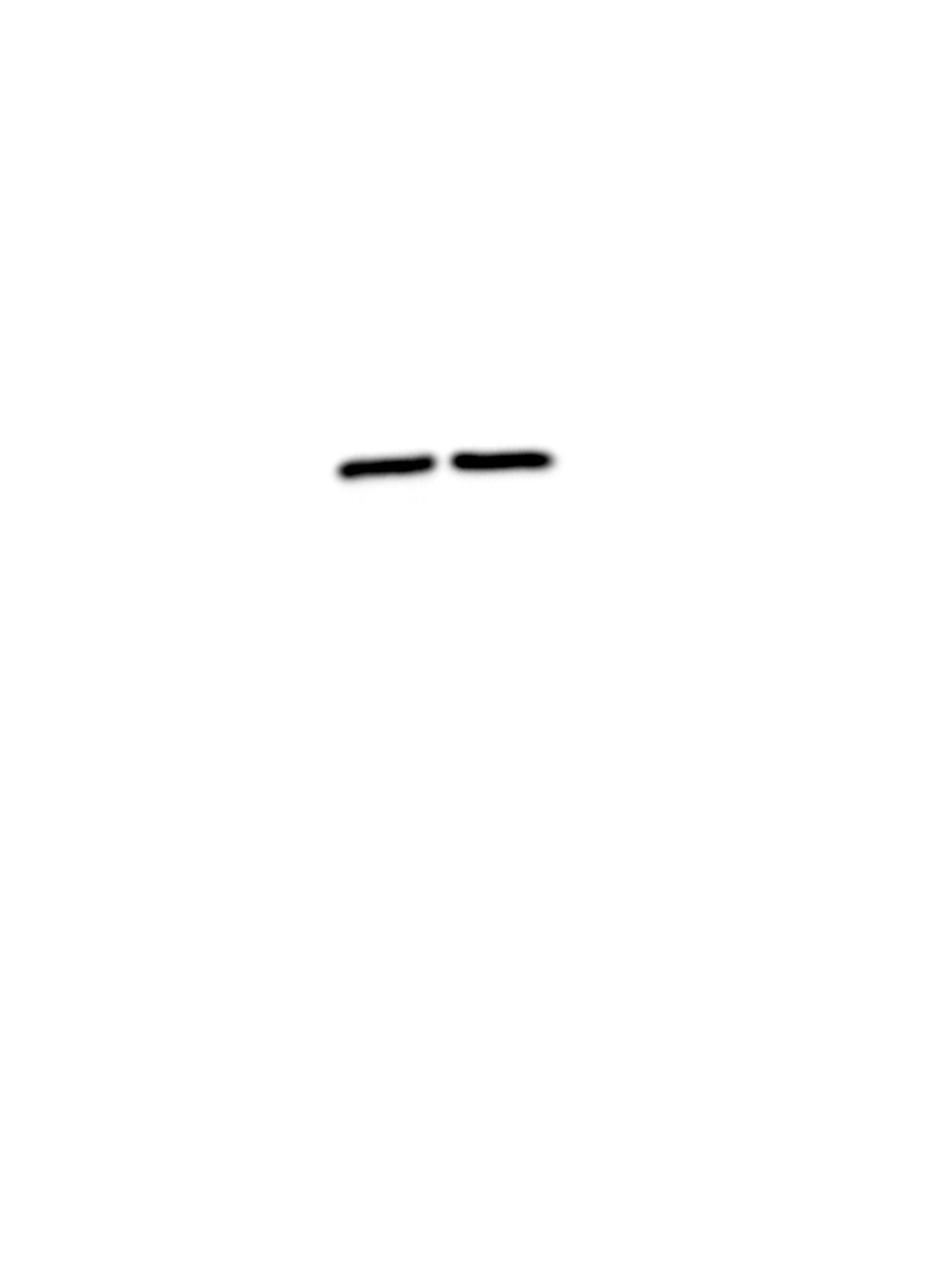


Fig.5B Western blot analysis of HOXD13-input


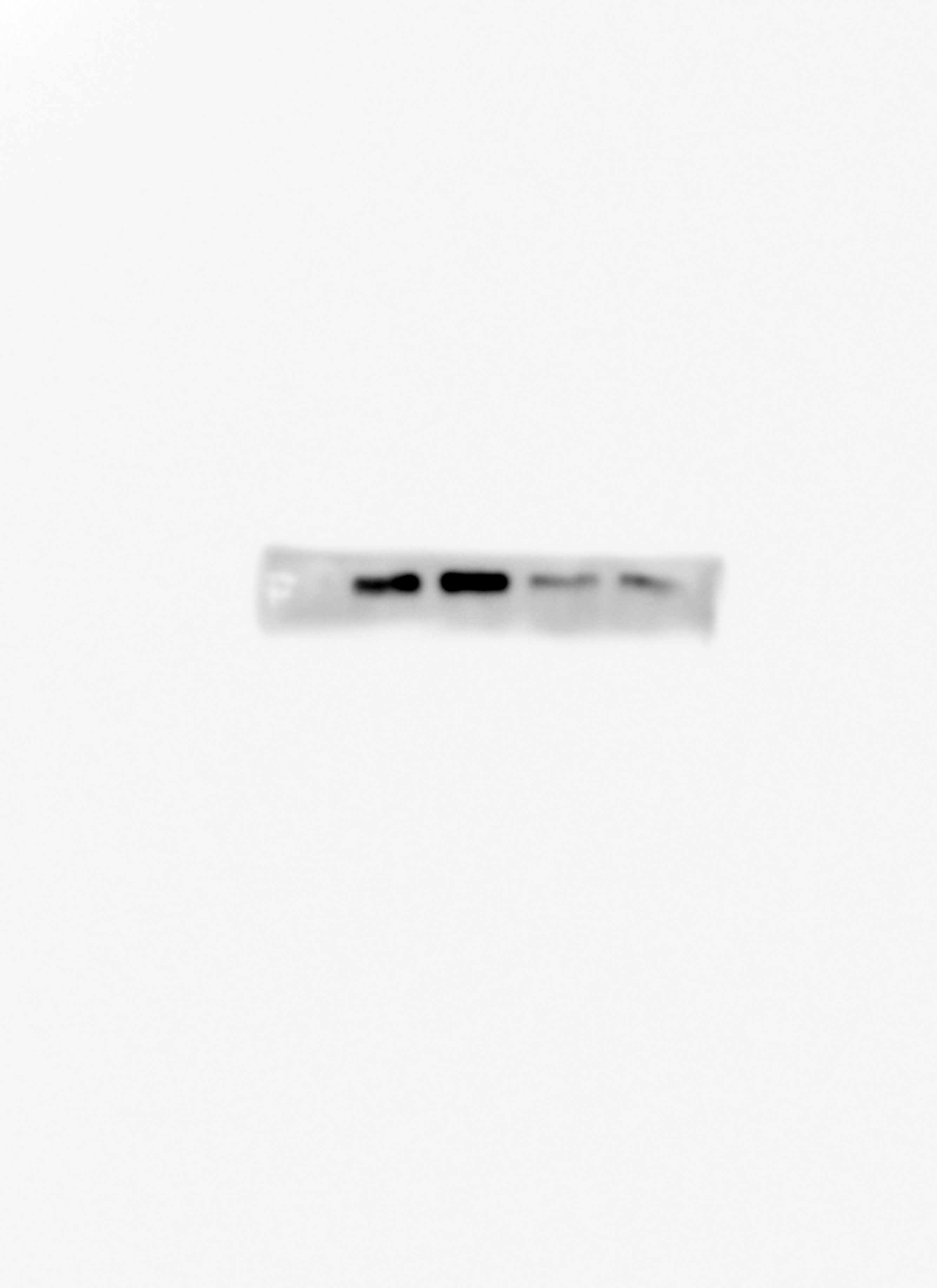


Fig.5C Western blot analysis of pSMAD5


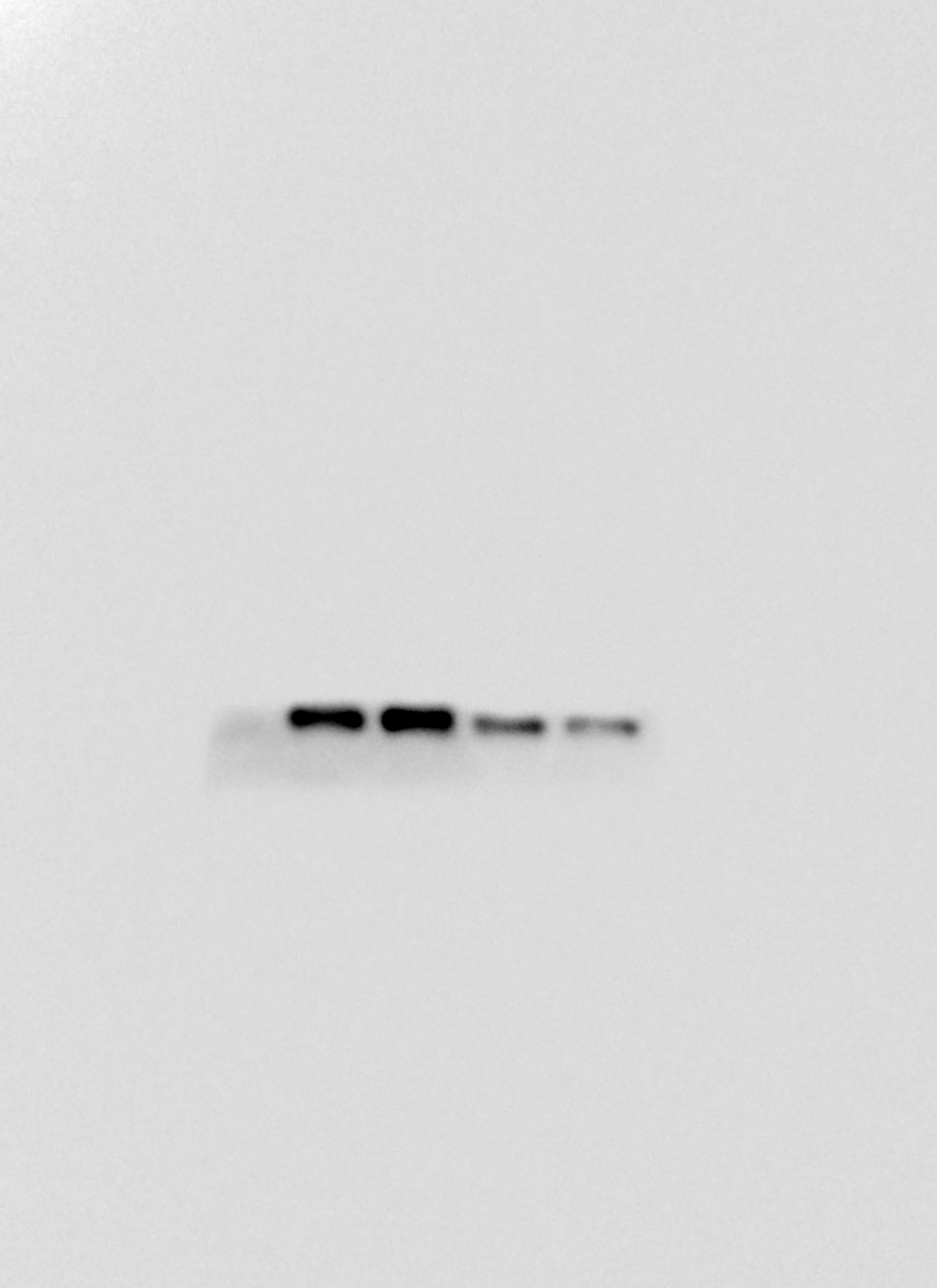


Fig.5C Western blot analysis of RANK


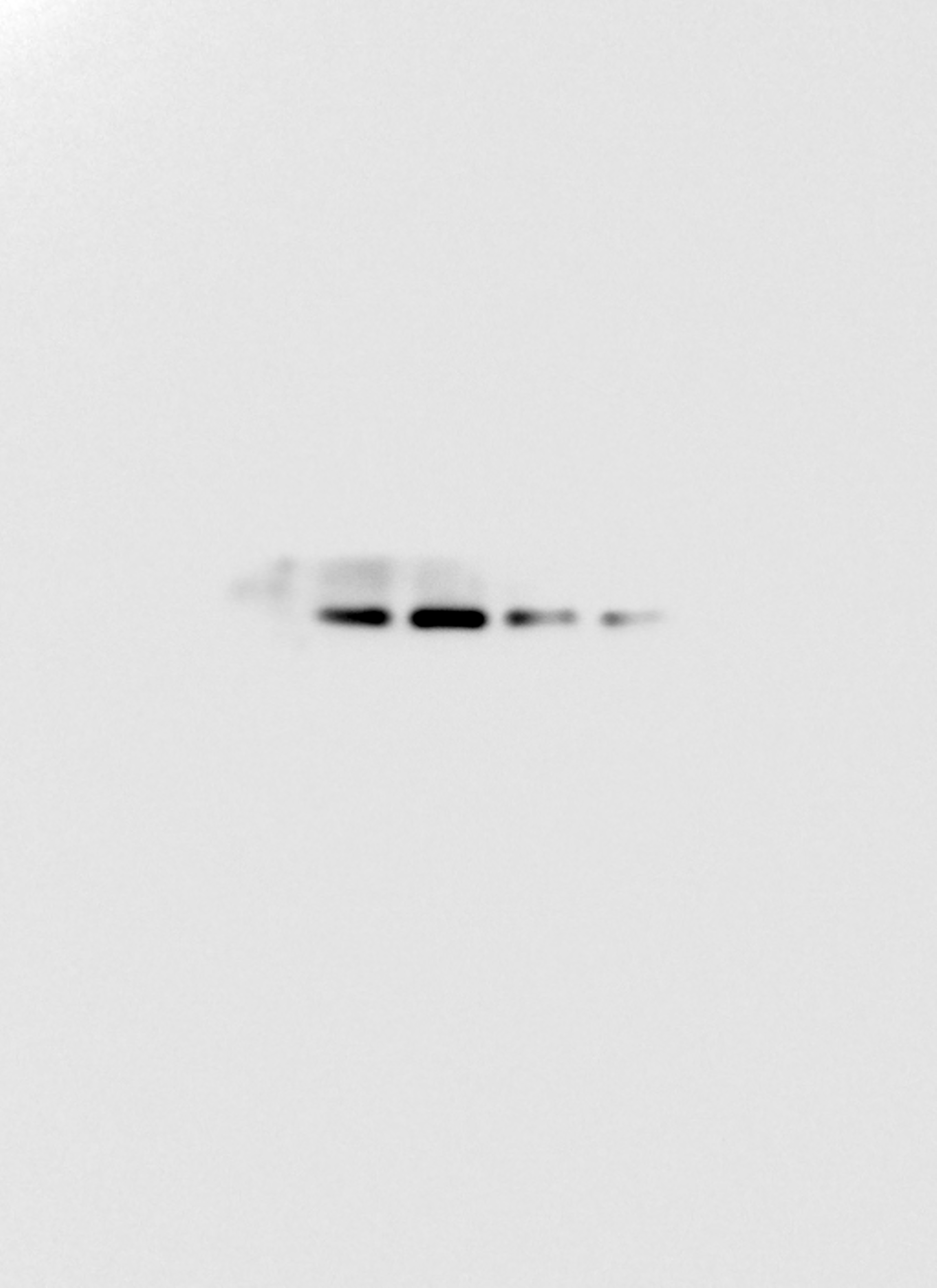


Fig.5C Western blot analysis of p65


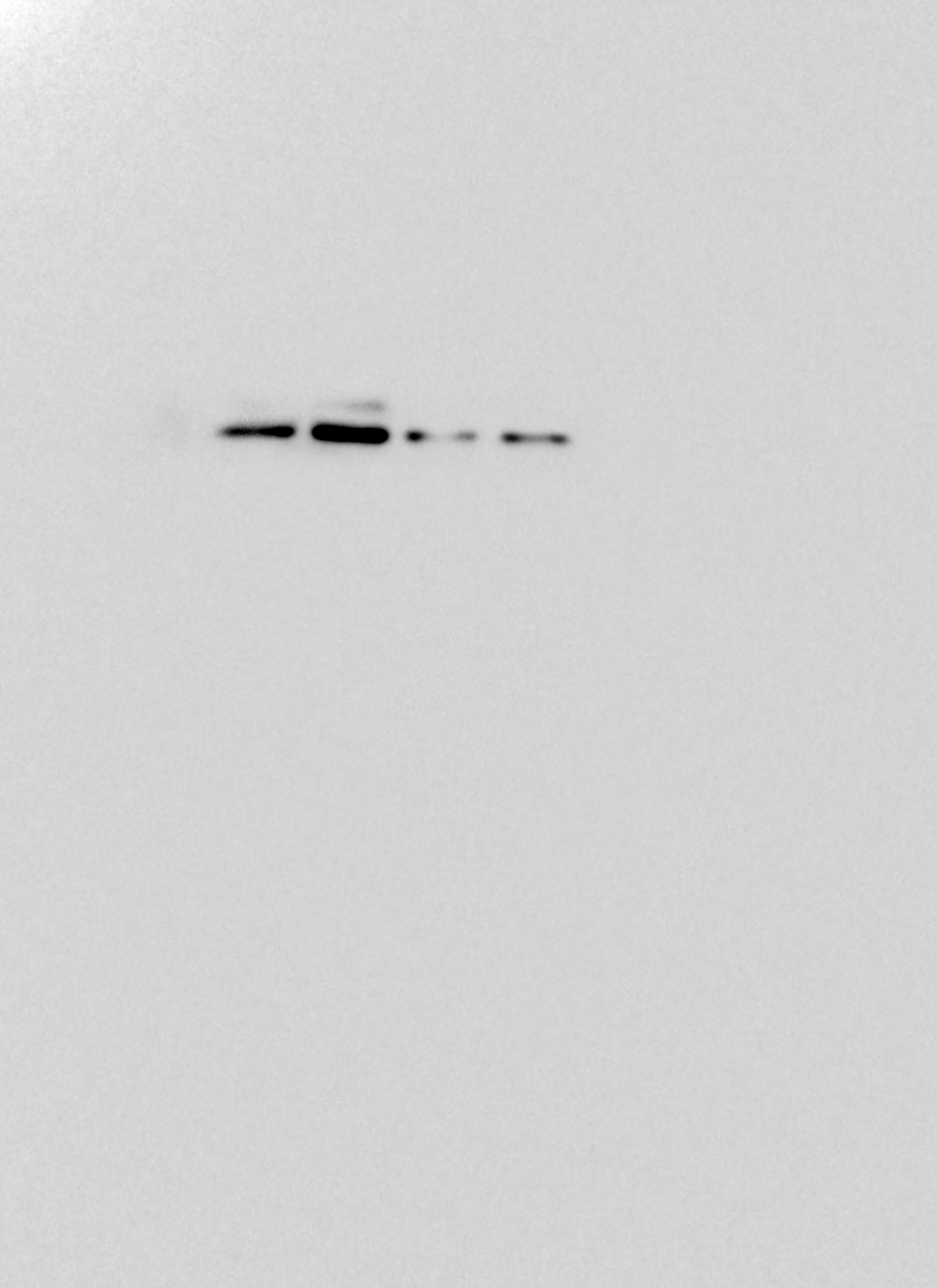


Fig.5C Western blot analysis of c-Fos


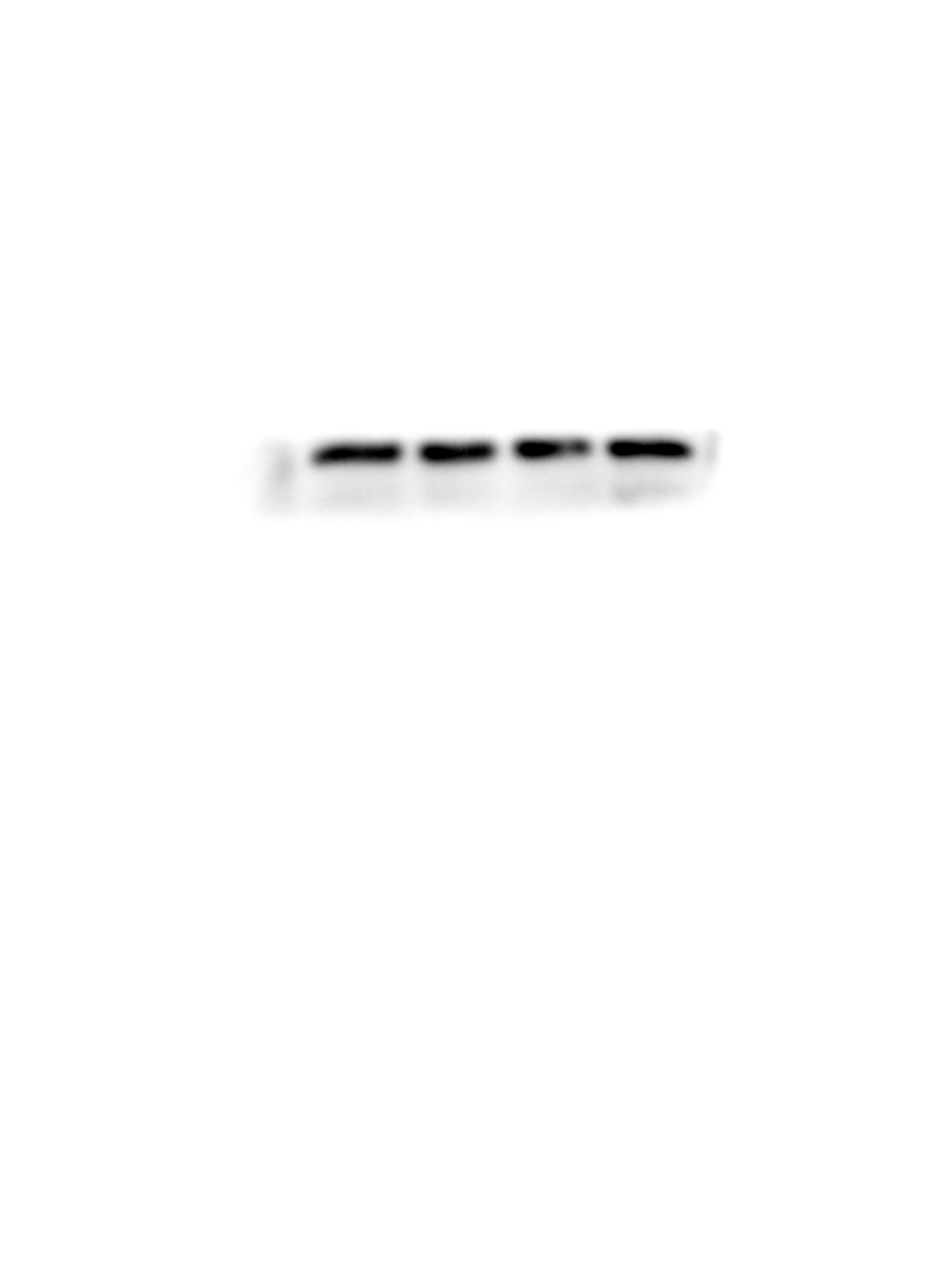


Fig.5C Western blot analysis of GAPDH
